# External evaluation of a deep learning-based approach for automated brain volumetry in patients with huntington’s disease

**DOI:** 10.1038/s41598-024-59590-7

**Published:** 2024-04-22

**Authors:** Robert Haase, Nils Christian Lehnen, Frederic Carsten Schmeel, Katerina Deike, Theodor Rüber, Alexander Radbruch, Daniel Paech

**Affiliations:** 1 Department of Neuroradiology, University Hospital Bonn, Venusberg-Campus 1, 53127 Bonn, Germany; 2 Department of Epileptology, University Hospital Bonn, Venusberg-Campus 1, 53127 Bonn, Germany

**Keywords:** Huntington's disease, Brain imaging

## Abstract

A crucial step in the clinical adaptation of an AI-based tool is an external, independent validation. The aim of this study was to investigate brain atrophy in patients with confirmed, progressed Huntington's disease using a certified software for automated volumetry and to compare the results with the manual measurement methods used in clinical practice as well as volume calculations of the caudate nuclei based on manual segmentations. Twenty-two patients were included retrospectively, consisting of eleven patients with Huntington's disease and caudate nucleus atrophy and an age- and sex-matched control group. To quantify caudate head atrophy, the frontal horn width to intercaudate distance ratio and the intercaudate distance to inner table width ratio were obtained. The software *mdbrain* was used for automated volumetry. Manually measured ratios and automatically measured volumes of the groups were compared using two-sample t-tests. Pearson correlation analyses were performed. The relative difference between automatically and manually determined volumes of the caudate nuclei was calculated. Both ratios were significantly different between the groups. The automatically and manually determined volumes of the caudate nuclei showed a high level of agreement with a mean relative discrepancy of − 2.3 ± 5.5%. The Huntington's disease group showed significantly lower volumes in a variety of supratentorial brain structures. The highest degree of atrophy was shown for the caudate nucleus, putamen, and pallidum (all *p* < .0001). The caudate nucleus volume and the ratios were found to be strongly correlated in both groups. In conclusion, in patients with progressed Huntington's disease, it was shown that the automatically determined caudate nucleus volume correlates strongly with measured ratios commonly used in clinical practice. Both methods allowed clear differentiation between groups in this collective. The software additionally allows radiologists to more objectively assess the involvement of a variety of brain structures that are less accessible to standard semiquantitative methods.

## Introduction

A large number of artificial intelligence-based software solutions are entering the diagnostic imaging market every year^1^. The technology has applications across the spectrum of radiology, particularly in characterization^2^, segmentation^3,4^, and detection tasks^5^. For radiologists, the integration into the daily workflow required in the face of increasing workloads is becoming a growing challenge—also due to the necessary assessment of the validity of various available software solutions. The diagnosis and follow-up of a large variety of neurodegenerative diseases are based on the assessment and evaluation of the volume loss of single or multiple brain regions. In addition to manual volume determinations using classical segmentation or the use of scoring systems and standardized measurement methods, an increasing number of automated software solutions are available to radiologists due to advances in machine learning. The software *mdbrain* (mediaire, Berlin, Germany) is an AI-based, CE-labelled, and commercially available software solution with approval as a medical device in the European Union. Among other features, it can be used for automated brain volumetry in patients with suspected neurodegenerative disease in addition to standard diagnostic procedures. The software has been used in scientific studies, including investigations of the impact of diseases such as autoimmune disorders^6^ and COVID-19^7^, or specific procedures^8^ on brain volumes.

Huntington’s disease (HD) is a progressive neurodegenerative disorder caused by an expanded cytosine-adenine-guanine-repeat in an allele of the huntingtin gene located on the short arm of chromosome four^9^. The prevalence of the mutation leading to an elongation of the polyglutamine strand in the huntingtin protein is about four to ten cases per 100,000 individuals in populations of Western European origin^10^. The mechanism of pathogenesis is complex and remains the subject of current research with an unclear role of the described aggregations of mutant huntingtin and other proteins^10^. Structural brain imaging using magnetic resonance imaging helps in guiding towards a possible diagnosis and is of importance in the subsequent assessment of progression. Here, however, semiquantitative measurements focus mostly on the basal ganglia, more precisely the heads of the caudate nuclei. Common ratios are the frontal horn width to intercaudate distance (FH/CC) ratio and the intercaudate distance to inner table width (CCI/IT) ratio^11,12^.

The aim of this study was to investigate the distribution of brain atrophy in patients with genetically confirmed HD and positive imaging findings of caudate nucleus atrophy using automated volumetry and to compare the results with the standard measurement methods used in clinical practice. The automatically determined volumes of the caudate nuclei were additionally validated using manual segmentations. Thus, this study serves as an external, independent evaluation of the present software utilized in our department using a small patient collective of a rare neurodegenerative disease.

## Methods

### Patients

By screening our in-house radiologic information system, all adult patients receiving MR imaging of the brain in our department since 2010 who met the inclusion criteria listed below were included.

Inclusion criteria were (a) imaging of the brain including a three-dimensional (3D), T1w sequence, (b) genetically confirmed HD diagnosis, and (c) imaging pathology in association with HD diagnosis in written report. All patients had positive imaging findings consistent with HD and were thus at an advanced stage of disease. This was necessary for the study to verify that present atrophy patterns are detected by the tested software. Eleven patients fulfilled the inclusion criteria and formed the HD group. Exclusion due to image artifacts (e.g., strong motion artifacts reducing the delineation of brain structures) or structural abnormalities (e.g., tumors in the area of the caudate nucleus) confounding the volume measurements was not necessary. No other exclusion criteria were applied. An age- and sex-matched control group of healthy patients was retrospectively composed that was examined with brain MR imaging in our department including an unenhanced, 3D T1w sequence. Patient characteristics, including age at time of examination, sex, disease duration, and age of symptom onset are summarized in Table 1.

**Table 1:** Patient characteristics.

Variable	Huntington group	Control group
No. included patients	11	11
Age (y)*	48.2 ± 13.0 (22–62)	48.3 ± 12.0 (26–60)
M/F	8/3	8/3
Field strength 1.5/3	3/8	0/11
Disease duration (y)*	3.1 ± 2.6 (1–10)	Not applicable
Age of symptom onset (y)*	44.6 ± 11.9 (21–58)	Not applicable
CAG triplets	45 ± 6 (38–61)	Not applicable

*Data are means ± standard deviation (range).

### MR imaging protocol

MR imaging of the brain was performed with a clinical 1.5T and 3T scanner (Achieva, Philips Healthcare, Best, The Netherlands). Eight HD patients were examined with a 3T scanner and three HD patients were examined with a 1.5T scanner. All patients in the control group received imaging with the 3T scanner. The standard imaging protocol of the HD patients included at least sagittal 3D T1w imaging, axial and coronal T2w imaging, axial FLAIR, axial DWI with ADC map, and SWI. In one of the included HD patients, the imaging protocol deviates slightly from this standard missing SWI. All control patients received at least an FLAIR, axial DWI with ADC map, and sagittal unenhanced 3D T1w imaging. The parameters of the 3D T1w sequence were TR in msec/TE in msec 8.7 ± 5.5 (6.6–25)/3.3 ± 0.5 (3.0–4.6) in the HD group and 7.3 ± 0 (7.3–7.4)/3.9 ± 0 (3.9–3.9) in the control group. 3D T1w images were acquired with a slice thickness of 1 mm and a resolution of at least 1 × 1 × 1 mm (*in-plane resolution* × *spacing between slices*). In one case of the HD group the slice thickness of the 3D T1w sequence was 2 mm.

### Quantitative analysis

**Figure 1:**
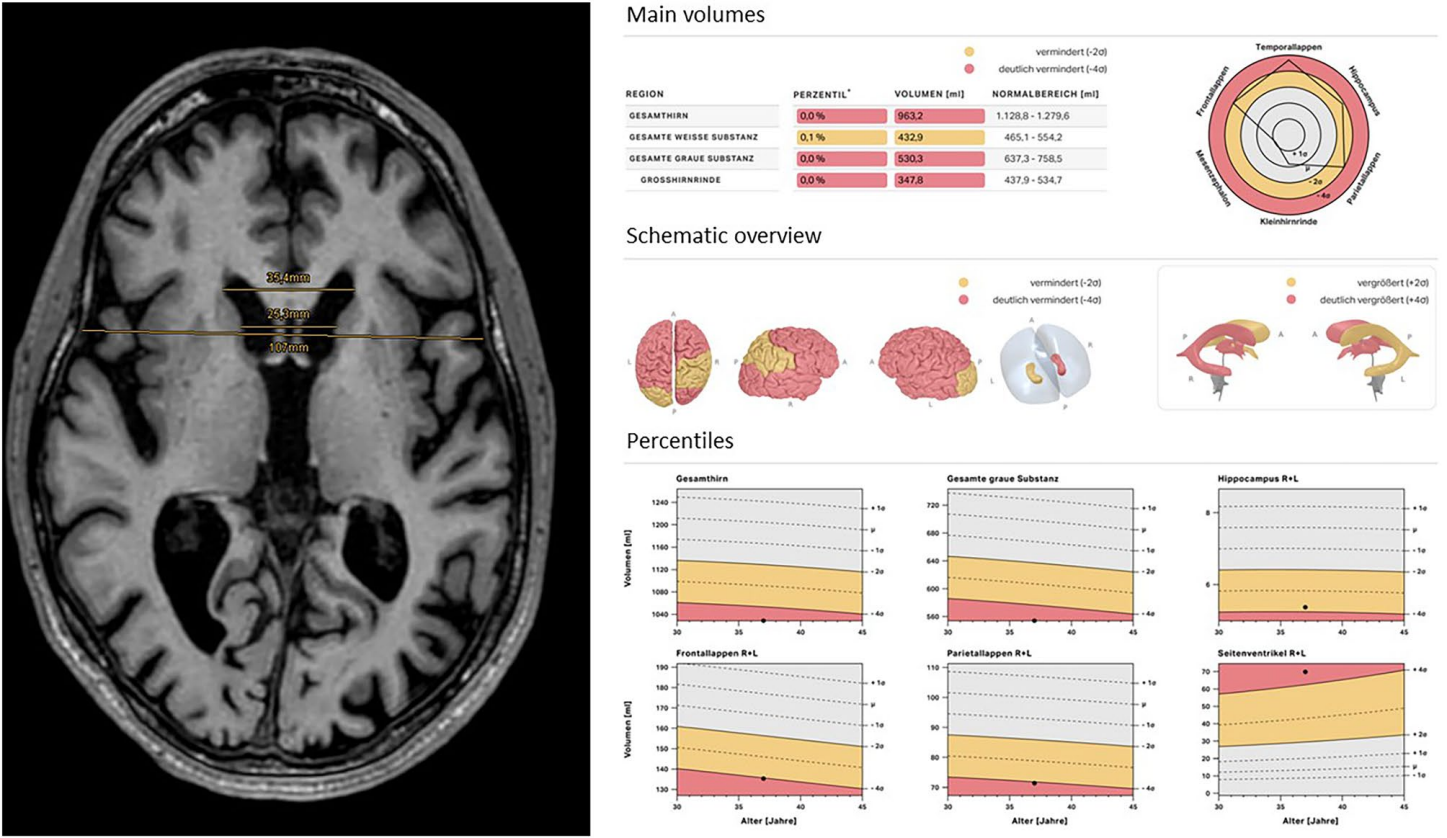
Sample excerpt of the software output (headings modified by the authors for translation from German) and manual measurements of the frontal horn width to intercaudate distance ratio and the intercaudate distance to inner table width ratio on axial planes obtained on the anterior commissure and posterior commissure line in a patient with severe atrophy of the caudate nucleus due to Huntington’s disease.

A retrospective reading session was performed by two readers in consensus (R.H. and D.P. with three and ten years’ experience in neuroimaging) to quantify caudate head atrophy by obtaining the FH/CC and CC/IT ratios on axial planes obtained on the anterior commissure and posterior commissure line. Additionally, the caudate nuclei were segmented using the open-source image computing platform *3D Slicer* (Version 5.6.0)^13^ by one reader (R.H.). Segmentations were checked by D.P. and used to calculate the respective volumes using the same platform. For obtaining the ratios, the distance between the lateral margins of the frontal horns, the distance between the inner table of the skull, and the distance between the caudate heads were measured on the plane where the caudate heads were closest. An example of the performed measurements can be seen in Fig. 1.

Subsequently, the 3D T1w sequence was sent to the *mdbrain* software (mediaire, Berlin, Germany), version 4.4.1, for automated volumetry. The determined volumes of all evaluated structures and the corresponding percentiles (based on an internal reference collective of the software) were saved and checked for plausibility. The measured structures were whole brain, whole white matter, whole gray matter, cerebral cortex, cerebellar cortex, frontal lobe, parietal lobe, precuneus, occipital lobe, temporal lobe, hippocampus, parahippocampal gyrus, entorhinal cortex, caudate nucleus, putamen, globus pallidum, thalamus, brainstem, mesencephalon, pons, lateral ventricle, third ventricle, and fourth ventricle. For paired structures, volumes were determined for each site.

The automated volumetry consists of the following steps:

1. Segmentation of the structures of interest. To this end, a custom deep learning segmentation model based on the U-Net architecture^14^ is employed. Before training of this model, the training data sets (balanced M/F, n = 2869 MRI scans with segmentation annotations obtained using a proprietary annotation process involving multiple human raters) were cropped to contain only the head and then resampled to a fixed size. To increase the model’s generalizability, various augmentation techniques were used, such as augmentation of contrast, resolution, rotation, and elastic deformation. The model was then trained on the preprocessed training data using the Adam variant of the stochastic gradient descent optimization algorithm^15^.
2. Determination of the volume of the structures of interest from the segmentation, by counting the number of voxels present in a segmentation mask and multiplying this count with the voxel volume.
3. Comparison to a reference population of healthy individuals (n = 6099, balanced M/F, mean age 41 ± 23 years, range 10–97 years, diverse image origin from Europe, the United States of America, Australia, and China) to determine percentiles while accounting for age, sex, and total intracranial volume.

As a Class IIb medical device, performance is validated internally for accuracy and repeatability. The supplier of the software confirmed that caudate volumetry passed all performance tests and was as reliable as volumetry of other small regions such as the hippocampus. However, these internal results were never published. Additionally, we could not find any publication that specifically investigated the capabilities of the software for caudate nucleus volumetry.

The software can run on a modern desktop PC (e.g., Intel i7 with 3 GHz and 16 GB RAM) with runtimes of about 10 min. Utilizing a GPU can significantly decrease runtimes to as low as one minute.

### Statistical analysis

Data were analyzed by using R version 4.2.1 (R Foundation for Statistical Computing, Vienna, Austria) and RStudio version 2022.07.01.554 (RStudio Team, Boston, MA). Installed packages were readxl, rstatix, pastecs, ggplot2, ggpubr, and dplyr. The a priori significance level was set to 0.05, and all reported *p*-values are two tailed. The assumption of a normal distribution of the FH/CC ratio, the CC/IT ratio, the structures’ volumes, and their percentiles was tested in each of the two groups using the Shapiro–Wilk test of normality. Two-sample t-tests were used to evaluate whether the true difference in means of the FH/CC ratio, the CC/IT ratio, and the volumes of the assessed structures between the HD group and control group was not equal to zero. Wilcoxon rank-sum tests were performed to compare the volumes of structures with significant results in the Shapiro–Wilk test, and to compare the percentiles of assessed structures of the groups provided by the software. *p*-values were adjusted using the Holm-Bonferroni method to prevent the problem of multiple comparisons (considering all 25 *p*-values). Pearson correlation analyses were performed to examine the correlation of the manually measured ratios and the automatically measured volumes. For paired structures, the mean value was used.

### Ethics approval

The study was approved by the Ethics Committee for Clinical Trials on Humans and Epidemiological Research with Personal Data of the Faculty of Medicine of the Rheinische Friedrich-Wilhelms-Universität Bonn (reference no. 118/22).

### Informed consent

This study did not require written informed consent due to the retrospective character.

## Results

Table 1 shows the patients characteristics including number of patients, age, sex, and field strength as well as disease duration and age of symptom onset. All cases could be processed by the software. The automatically determined volumes of the caudate nuclei showed a high level of agreement with the manually determined volumes with a mean relative discrepancy of − 2.3 ± 5.5% (range of − 12.1–7.9%) (HD group: − 2.7 ± 4.9%; Control group: − 1.8 ± 6.0%). The Shapiro–Wilk test of normality indicated that the null hypothesis of a normal distribution could be accepted for all ratios and volumes in both groups, except for the volumes of the parahippocampal gyrus in the HD group and the volumes of the lateral ventricle, parietal lobe, and temporal lobe in the control group (HD group: FH/CC, *p* = 0.89; CC/IT, *p* = 0.14; whole brain, *p* = 0.89; caudate nucleus, *p* = 0.16; Control group: FH/CC, *p* = 0.07; CC/IT, *p* = 0.75; whole brain, *p* = 0.27; caudate nucleus, *p* = 0.34). The percentiles of the HD group could not be considered normally distributed in the majority of structures.

**Figure 2:**
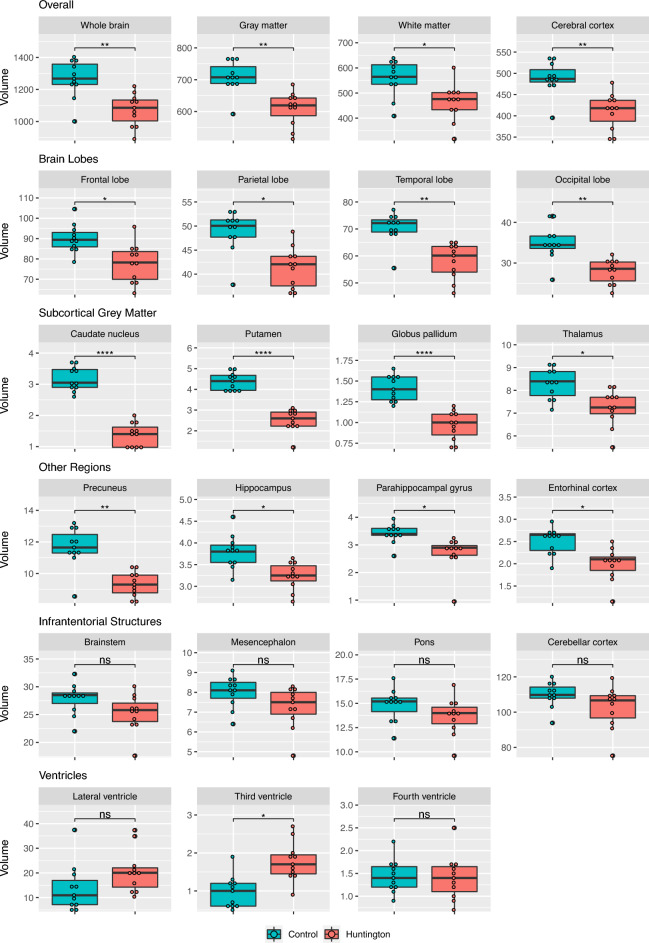
Box-and-whisker plots of the automated volume measurements of all structures in the Huntington and control group. Volume in ml. Used convention for symbols indicating statistical significance: ns: *p* > .05; *: *p* ≤ .05; **: *p* ≤ .01; ***: *p* ≤ .001; ****: *p* ≤ .0001.

The mean FH/CC and CC/IT ratios were significantly different between the HD and control group (FH/CC:* p* < 0.0001, HD group: 1.83 ± 0.27, Control group: 3.18 ± 0.54; CC/IT: *p* < 0.0001, HD group: 0.17 ± 0.03, Control group: 0.09 ± 0.02). Analysis of the results of the automated brain volumetry showed significantly lower volumes of the whole brain, whole grey matter, whole white matter, cerebral cortex, caudate nucleus, putamen, globus pallidus, thalamus, frontal lobe, parietal lobe, temporal lobe, occipital lobe, precuneus, hippocampus, parahippocampal gyrus, and entorhinal cortex in the HD group compared with the control group. The highest levels of significance were shown for the caudate nucleus, putamen, and globus pallidus (all *p* < 0.0001). The mean, standard deviation, as well as original and adjusted *p*-values of some of the many structures analyzed can be found in Table 2. The results for all brain volumes are reported in the Supplementary Table S1. Figure 2 shows Box-and-whisker plots for all assessed structures.

**Table 2:** Mean volume, Standard deviation (SD), Results of Two-sample t-tests, Statistical significance, and Number of cases marked as potentially pathologic by the software of selected volumes.

Variable	Mean ± SD (Huntington), volume in ml	Mean ± SD (Control), volume in ml	Two-sample t-test, *p*-value	Adjusted *p*-value (Holm)^a^	Statistical Significance^b^	Marked cases (out of 11), Huntington/Control group^c^
FH/CC	1.83 ± 0.27	3.18 ± 0.54	< .0001	< .0001	****	10/ 0
CC/IT	0.17 ± 0.03	0.09 ± 0.02	< .0001	< .0001	****	11/ 0
Whole brain	1073 ± 101	1266 ± 118	.0005	.008	**	7/ 0
Whole white matter	463 ± 74	558 ± 72	.006	.045	*	4/ 0
Whole gray matter	609 ± 53	708 ± 50	.0002	.004	**	7/ 0
Cerebral cortex	411 ± 42	488 ± 39	.0002	.004	**	7/0
Cerebellar cortex	103 ± 12	110 ± 7	.11	.32	ns	1/0
Occipital lobe	27.9 ± 3.0	35.0 ± 4.3	.0003	.005	**	6/0
Caudate nucleus	1.4 ± 0.4	3.2 ± 0.4	< .0001	< .0001	****	11/0
Putamen	2.5 ± 0.6	4.4 ± 0.4	< .0001	< .0001	****	11/0
Globus pallidus	1.0 ± 0.2	1.4 ± 0.2	< .0001	< .0001	****	11/0
Thalamus	7.2 ± 0.8	8.3 ± 0.7	.003	.03	*	5/1
Brainstem	25.3 ± 3.3	27.8 ± 2.8	.06	.32	ns	3/0
Third ventricle	1.8 ± 0.5	1.0 ± 0.4	.0008	.01	*	11/1

^a^Application of the Holm correction (Considering all 25 *p*-values).

^b^Used convention for symbols indicating statistical significance: ns: *p* > .05; *: *p* ≤ .05; **: *p* ≤ .01; ***: *p* ≤ .001; ****: *p* ≤ .0001.

^c^FH/CC and CC/IT: Number of cases with a pathological ratio assuming a ratio of 0.09–0.12 for CC/IT and of 2.2–2.6 for FH/CC as normal^12^. Other: Number of cases marked by the software as potentially pathologic with a difference of the volume from the internal reference collective of the software by more than two standard deviations.

Results of all structures can be found in Supplementary Table S1.

The software compares the determined volumes with an internal reference group and provides a percentile value in addition to the volume. The comparison of the percentiles of both groups yielded similar results (see also Supplementary Table S2). A decreased volume of both caudate nucleus, putamen, and globus pallidus by at least two standard deviations compared with the internal reference group of the software was present in all cases of the HD group and in no case of the study control group (see also Table 2). The median and interquartile range as well as all original and adjusted *p*-values of the Wilcoxon rank-sum tests are reported for all brain volumes in Supplementary Table S2. Box-and-whisker plots of the percentiles for all assessed structures are shown in Supplementary Fig. S1.

**Figure 3:**
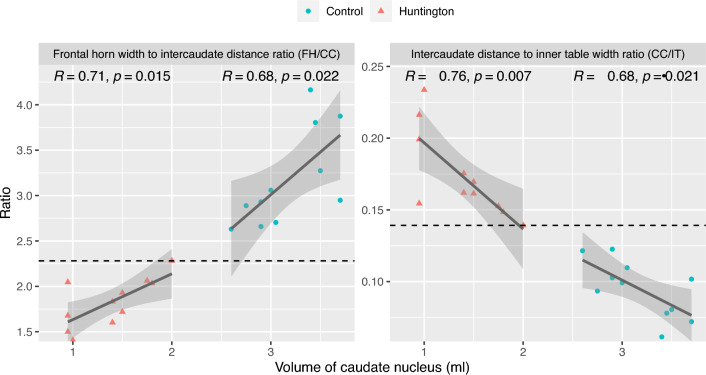
Scatterplot of the caudate nucleus volume and the values of the frontal horn width to intercaudate distance ratio and the intercaudate distance to inner table width ratio for each group. Dashed line marking the most extreme value of the Huntington's group in the direction of the control group as cut-off value.

The volume of the caudate nucleus and the measured ratios (FH/CC and CC/IT) were found to be strongly correlated in both groups (HD group: FH/CC: *r*(9) = 0.71, *p* = 0.015; CC/IT: *r*(9) = − 0.76, *p* = 0.007; Control group: FH/CC: *r*(9) = 0.68, *p* = 0.022; CC/IT: *r*(9) = − 0.68, *p* = 0.021) (Fig. 3). Both ratios and the automatically determined caudate nucleus volume allowed clear differentiation between groups in this collective, with a cutoff value of 2.28 for the FH/CC ratio, 0.139 for the CC/IT ratio, and 2.0 ml for the mean volume of the caudate nuclei.

## Discussion

In this monocentric study of patients with confirmed, progressed HD and an associated imaging pathology in written report, it was shown that the caudate nucleus volume automatically determined by the tested deep learning-based software shows a high level of agreement with the manually determined volumes and correlates strongly with measured ratios commonly used in clinical practice. With both the volume and the ratios, a clear identification of patients with advanced HD was possible.

The values of FH/CC and CC/IT ratios for both groups are consistent with those reported in the literature for adult patients^11^. The automated volumetry of the patients’ brains showed broad atrophy of supratentorial structures in the HD group, with emphasis not only in the caudate nucleus but also in the putamen, globus pallidus, temporal lobe, precuneus, and occipital lobe. The significance of the determined volume differences between the study groups remained when comparing the percentile values output by the software using an internal reference group. This controlled for the influence of possible differences in intracranial volume between the HD group and control group. The output of percentiles and their classification in terms of standard deviations from the stored reference collective of the software enables assessments of the determined volumes of individual cases without a control group in everyday clinical practice.

Our findings of regional atrophies are consistent with other structural imaging studies in which the subcortical structures showed the earliest^16^ and most severe atrophy^17,18^. The known involvement of white matter^17^ in the disease and the accentuation of atrophy of posterior cortical structures^17,19–21^ and the relative preservation of cerebellar cortex^19^ predescribed in other studies was also evident by automated volumetry in our study. Volumetric analyses of HD patients using the open-source software *FreeSurfer* (http://surfer.nmr.mgh.harvard.edu/) showed similar atrophy patterns with atrophy prominence in striatal structures and the occipital lobe^22^.

Our study serves as an external evaluation of the tested software for automated brain volumetry using a study sample with a rare neurodegenerative disease. This is a crucial step in the adaptation of an artificial intelligence-based tool in everyday clinical practice^23^. While the detectability of intracranial aneurysms detection has already been investigated in a clinical setting^24^, the published evidence on brain volumetry employing the investigated software is limited to a few publications^6–8^ and conference abstracts^25–27^.

Our study has limitations.

First, the number of patients was relatively small. This is due to the rarity of the disease studied. However, the study sample includes all patients with HD who received in-hospital imaging with a comparable imaging protocol from 2010 to present. Nonetheless, we were able to demonstrate that the tested software allows reliable volume determination for the identification of patients with basal ganglia atrophy.

Second, in contrast to the control group, three patients in the HD group were examined at a field strength of 1.5T instead of 3T, introducing the possibility of a volume difference bias. However, as the determined volumes at 3T are expected to be lower than at 1.5T due to the improved tissue-CSF contrast,^28,29^ such a bias would hinder rather than assist the detection of differences between the groups in this study. Given the low proportion of patients with 1.5T in the HD group, the significant differences in measured volumes between the groups, and the high agreement with manually determined volumes at both field strengths, we consider this bias to be negligible.

Third, this is a retrospective study. Prospective investigations of the use of the software would provide further insight regarding the impact on diagnostic decisions and time efficiency.

Fourth, all patients had positive imaging findings consistent with HD and were thus at an advanced stage of disease. Although this was necessary for the study to verify that present atrophy patterns are detected by the tested software, it remains unclear whether automated volumetry using the software allows earlier detection of atrophy pattern in HD. This question should be addressed in future studies.

## Conclusions

In conclusion, the software allows radiologists to objectively assess the involvement of a variety of brain structures in patients with HD that are less accessible to standard semiquantitative methods. Our data suggests that the software can help in providing a more detailed assessment of the impact of HD on the individual patient. The significantly lower barrier in the application compared to most script-based, open-source software could allow a broad application in the clinical setting outside of scientific research. In particular for follow-up examinations, the objectivity could have additional value.

### Supplementary Information

Supplementary Information.

## Data Availability

*Data*: The imaging data and datasets generated during during the current study are not publicly available due to data protection laws and the retrospective character of the study, which did not require written informed consent, but are available from the corresponding author on reasonable request.

## Abbreviations

3D: Three-dimensional
CCI/IT: Intercaudate distance to inner table width
FH/CC: Frontal horn width to intercaudate distance
HD: Huntington’s disease

## Code availability

The tested program is a commercially available product.
