# Supplementary material for: External evaluation of a deep learning-based approach for automated brain volumetry in patients with huntington’s disease
Source: Sci Rep. 2024 Apr 22;14:9243. doi: 10.1038/s41598-024-59590-7 (PMC11035562; doi:10.1038/s41598-024-59590-7)

Supplements

Supplementary material of the article *External Evaluation of a Deep Learning-based Approach For Automated Brain Volumetry in Patients with Huntington's Disease* published in *Scientific Reports*.

Author names:

Robert Haase[1], Nils Christian Lehnen[1], Frederic Carsten Schmeel[1], Katerina Deike[1], Theodor Rüber[2], Alexander Radbruch[1], Daniel Paech[1*]

Affiliations:

[1]Department of Neuroradiology, University Hospital Bonn, Venusberg-Campus 1, 53127, Bonn, Germany

[2]Department of Epileptology, University Hospital Bonn, Venusberg-Campus 1, 53127, Bonn, Germany

***Corresponding author:**

PD Dr. Daniel Paech, M.D., Ph.D., Department of Neuroradiology, University Hospital Bonn, Venusberg-Campus 1, 53127, Bonn, Germany, E-mail: daniel.paech@ukbonn.de, Phone: +49 228–287 31092

Supplementary Tab. S1 Mean volume, Standard deviation (SD), Results of Two-sample t-tests, Statistical significance, and Number of cases marked as potentially pathologic by the software.

**Variable**	**Mean ± SD (Huntington), volume in ml**	**Mean ± SD (Control), volume in ml**	**Two-sample t-test, p-value**	**Adjusted p-value (Holm)[a]**	**Statistical Significance[b]**	**Marked cases (out of 11), Huntington/ Control group[c]**
**FH/CC**	1.83 ± 0.27	3.18 ± 0.54	<.0001	<.0001	****	10/ 0
**CC/IT**	0.17 ± 0.03	0.09 ± 0.02	<.0001	<.0001	****	11/ 0
**whole brain**	1073 ± 101	1266 ± 118	.0005	.008	**	7/ 0
**whole white matter**	463 ± 74	558 ± 72	.006	.045	*	4/ 0
**whole gray matter**	609 ± 53	708 ± 50	.0002	.004	**	7/ 0
**cerebral cortex**	411 ± 42	488 ± 39	.0002	.004	**	7/ 0
**cerebellar cortex**	103 ± 12	110 ± 7	.11	.32	ns	1/ 0
**frontal lobe**	77.9 ± 9.6	90.0 ± 7.0	.003	.03	*	4/ 0
**parietal lobe**	41.4 ± 4.2	48.9 ± 4.3	.0008[d]	.01	*	5/ 0
**precuneus**	9.3 ± 0.8	11.7 ± 1.3	<.0001	.002	**	3/ 0
**occipital lobe**	27.9 ± 3.0	35.0 ± 4.3	.0003	.005	**	6/ 0
**temporal lobe**	58.2 ± 6.6	70.4 ± 5.7	.0001[d]	.002	**	3/ 0
**hippocampus**	3.2 ± 0.3	3.8 ± 0.4	.001	.015	*	1/ 0
**parahippocampal gyrus**	2.7 ± 0.6	3.4 ± 0.4	.001[d]	.01	*	3/ 0
**entorhinal cortex**	2.0 ± 0.4	2.5 ± 0.3	.002	.02	*	2/ 0
**caudate nucleus**	1.4 ± 0.4	3.2 ± 0.4	<.0001	<.0001	****	11/ 0
**putamen**	2.5 ± 0.6	4.4 ± 0.4	<.0001	<.0001	****	11/ 0
**globus pallidus**	1.0 ± 0.2	1.4 ± 0.2	<.0001	<.0001	****	11/ 0
**thalamus**	7.2 ± 0.8	8.3 ± 0.7	.003	.03	*	5/ 1
**brainstem**	25.3 ± 3.3	27.8 ± 2.8	.06	.32	ns	3/ 0
**mesencephalon**	7.3 ± 1.0	8.0 ± 0.8	.07	.32	ns	3/ 0
**pons**	13.7 ± 1.9	14.9 ± 1.7	.14	.32	ns	2/ 0
**lateral ventricle**	20.6 ± 8.7	13.8 ± 9.6	.05[d]	.28	ns	5/ 2
**third ventricle**	1.8 ± 0.5	1.0 ± 0.4	.0008	.01	*	11/ 1
**fourth ventricle**	1.4 ± 0.5	1.4 ± 0.4	.88	.88	ns	1/ 1

[a]Application of the Holm correction

[b]Used convention for symbols indicating statistical significance: ns: $p > .05$; *: $p \le .05$; **: $p \le .01$; ***: $p \le .001$; ****: $p \le .0001$

[c]FH/CC and CC/IT: Number of cases with a pathological ratio assuming a ratio of 0.09-0.12 for CC/IT and of 2.2-2.6 for FH/CC as normal. Other: Number of cases marked by the software as potentially pathologic with a difference of the volume from the internal reference collective of the software by more than two standard deviations.

[d]Wilcoxon rank-sum tests were performed to compare the volumes of structures with significant results in the Shapiro-Wilk test.

Supplementary Tab. S2 Median and Interquartile range (IQR) of percentiles of the volumetrized structures, Results of Wilcoxon rank sum-test, and Statistical Significance

**Variable**	**Median, IQR (Huntington), percentile**	**Median, IQR (Control), percentile**	**Wilcoxon rank-sum test, p-value**	**Adjusted p-value (Holm)[a]**	**Statistical Significance[b]**
**whole brain**	0.7, 4.8	77.1, 17.6	<.0001	.002	**
**whole gray matter**	0.5, 4.4	37.8, 29.9	.001	.01	*
**whole white matter**	4.5, 12.6	89.5, 36.7	.001	.01	*
**cerebral cortex**	1.0, 4.9	39.1, 38.6	.001	.01	*
**cerebellar cortex**	61.1, 40.1	73.4, 28.7	.44	1	ns
**frontal lobe**	9.3, 31.0	59.0, 42.6	.02	.12	ns
**parietal lobe**	2.3, 6.8	31.2, 24.6	.002	.02	*
**precuneus**	4.8, 12.4	50.8, 27.6	.0004	.005	**
**occipital lobe**	0.9, 2.2	33.5, 24.1	.0001	.003	**
**temporal lobe**	3.2, 10.6	49.1, 29.9	.002	.01	*
**hippocampus**	8.2, 8.2	45.3, 31.9	<.0001	.0008	***
**parahippocampal gyrus**	4.8, 22.6	62.4, 34.1	.0002	.003	**
**entorhinal cortex**	8.9, 11.8	65.1, 37.8	.0002	.003	**
**caudate nucleus**	0, 0.1	32.7, 42.4	<.0001	.001	**
**putamen**	0, 0	26.5, 32.8	<.0001	.0008	***
**globus pallidus**	0, 0.1	25.9, 37.7	<.0001	.001	**
**thalamus**	1.6, 8.7	38.7, 29.1	.0004	.005	**
**brainstem**	22.9, 70.2	59.2, 30.2	.27	1	ns
**mesencephalon**	40.2, 49.2	59.6, 21.6	.09	.44	ns
**pons**	40.2, 63.9	49.9, 38.9	.37	1	ns
**lateral ventricle**	95.3, 7.6	52.8, 42.8	.004	.03	*
**third ventricle**	99.9, 0.2	73.3, 36.9	.0002	.003	**
**fourth ventricle**	50.8, 52.6	50.8, 44.6	.79	1	ns

[a]Application of the Holm correction. The unequal distribution of percentile volumes between groups should be noted. Due to ties, exact p-values cannot be calculated for some volumes. Therefore, an exact comparison is not reasonable.

[b]Used convention for symbols indicating statistical significance: ns: $p > .05$; *: $p \le .05$; **: $p \le .01$; ***: $p \le .001$; ****: $p \le .0001$.

Supplementary Fig. S1 Box-and-whisker plots of the percentiles of all structures automatically volumetrized in the Huntington and control group. Used convention for symbols indicating statistical significance: ns: $p > .05$; *: $p \le .05$; **: $p \le .01$; ***: $p \le .001$; ****: $p \le .0001$

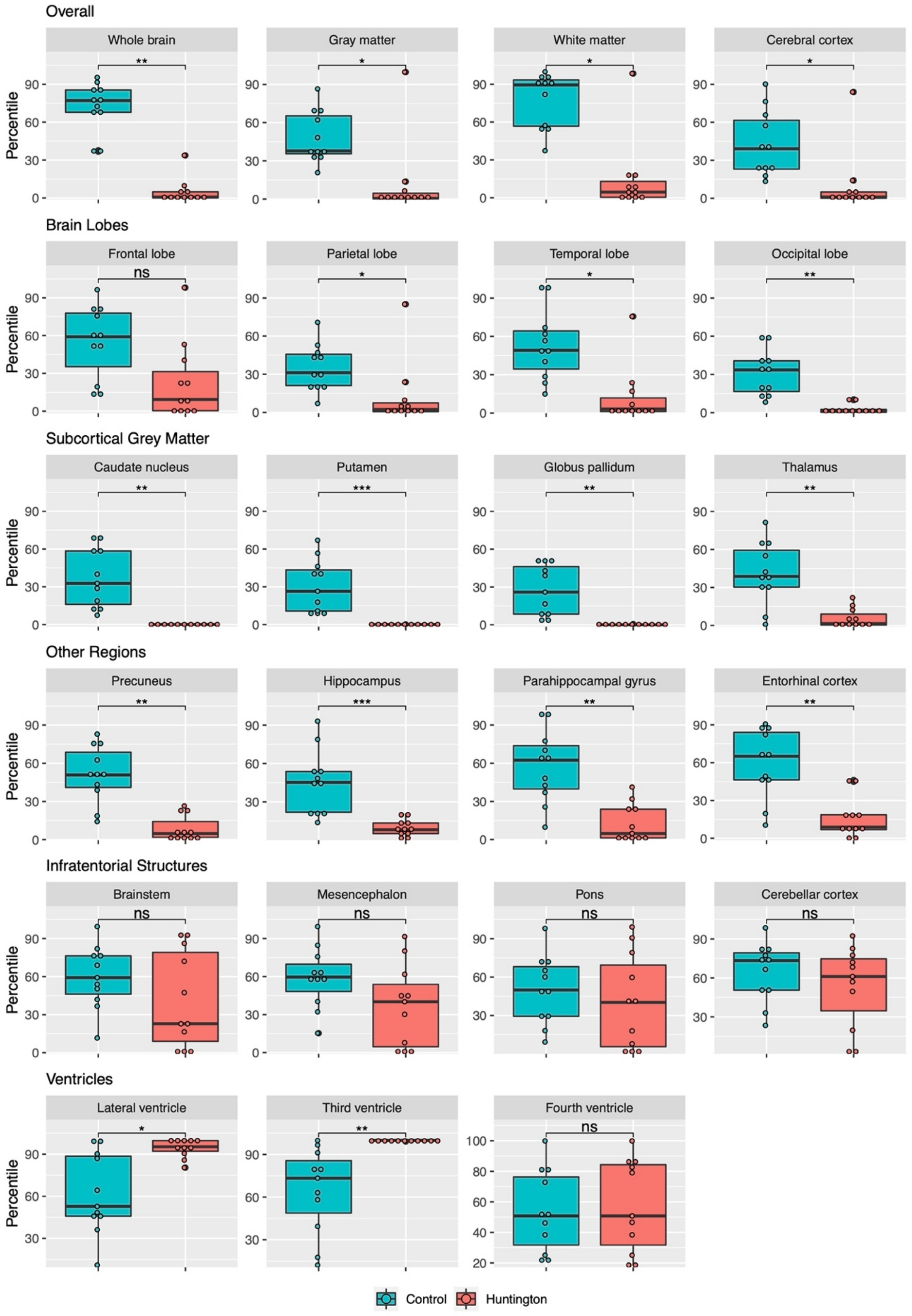

Supplement: Supplementary file 1 — Supplementary Information. [file 41598_2024_59590_MOESM1_ESM.pdf]
